# Isoallopregnanolone Inhibits Estrus Cycle-Dependent Aggressive Behavior

**DOI:** 10.3390/biom13061017

**Published:** 2023-06-20

**Authors:** Torbjörn Bäckström, Sara K. S. Bengtsson, Jessica Sjöstedt, Evgenya Malinina, Maja Johansson, Gianna Ragagnin, Karin Ekberg, Per Lundgren

**Affiliations:** 1 Umeå Neurosteroid Research Center, Department of Clinical Science, Umeå University, SE-901 85 Umea, Sweden; 2 Asarina Pharma AB, Fogdevreten 2, SE-171 65 Solna, Sweden

**Keywords:** estrus cycle, estrus cycle-dependent aggression, diestrus, estrus, allopregnanolone, isoallopregnanolone, Wistar rats, resident/intruder test

## Abstract

Among female rats, some individuals show estrus cycle-dependent irritability/aggressive behaviors, and these individual rats may be used as a model for premenstrual dysphoric disorder (PMDD). We wanted to investigate if these behaviors are related to the estrus cycle phase containing moderately increased levels of positive GABA-A receptor-modulating steroids (steroid-PAM), especially allopregnanolone (ALLO), and if the adverse behavior can be antagonized. The electrophysiology studies in this paper show that isoallopregnanolone (ISO) is a GABA-A-modulating steroid antagonist (GAMSA), meaning that ISO can antagonize the agonistic effects of positive GABA-A receptor-modulating steroids in both α1β2γ2L and α4β3δ GABA-A receptor subtypes. In this study, we also investigated whether ISO could antagonize the estrus cycle-dependent aggressive behaviors in female Wistar rats using a resident–intruder test. Our results confirmed previous reports of estrus cycle-dependent behaviors in that 42% of the tested rats showed higher levels of irritability/aggression at diestrus compared to those at estrus. Furthermore, we found that, during the treatment with ISO, the aggressive behavior at diestrus was alleviated to a level comparable to that of estrus. We noticed an 89% reduction in the increase in aggressive behavior at diestrus compared to that at estrus. Vehicle treatment in the same animals showed a minimal effect on the diestrus-related aggressive behavior. In conclusion, we showed that ISO can antagonize Steroid-PAM both in α1β2γ2L and α4β3δ GABA-A receptor subtypes and inhibit estrus cycle-dependent aggressive behavior.

## 1. Introduction

Among female rats, some individuals show estrus cycle-dependent aggression. This has been shown in the otherwise non-aggressive Wistar strain in up to 30% of the population [1] and in up to 60% of the population when the aggression was heightened by pseudopregnancy [2]. Compared to estrus, during which there was almost no aggression, the rats showed increased aggressive behavior at diestrus.

Depending on the estrus cycle phases, the levels of progesterone and associated γ-amino-butyric acid type A (GABA-A) receptor-modulating steroids (steroid-PAM) vary. They are moderately increased at early diestrus and decreased at late diestrus [3]. From late proestrus to estrus, the levels of progesterone and steroid-PAMs hit a high peak, which rapidly drops again at late estrus. The steroid-PAMs modulate the effect of GABA on the GABA-A receptor [4]. The most potent steroid-PAMs are allopregnanolone (ALLO; 3α-OH-5α-pregnan-20-one) and tetrahydro-deoxycorticosterone (THDOC), which are progesterone and deoxycorticosterone metabolites. They both strongly enhance the GABAergic effect on synaptic receptors, and also, have an effect on extra synaptic receptors without GABA being present [5,6]. Generally, this effect is sedative and like that of benzodiazepines, barbiturates, and alcohol. However, in some individuals, a paradoxical effect of increased aggression is produced by moderately increased levels [7,8,9]. This is similar to the effect of all types of positive GABA-A receptor modulators [8]. This is most likely due to the disinhibition of related neuronal activity and leads to behaviors that are seen in, for example, the case of estrus cycle-dependent aggression among female rats.

These symptoms of aggression or irritability and the linkage to the ovarian cycle are similar to the human condition, premenstrual dysphoric disorder (PMDD). PMDD occurs in a subgroup of women (3–8%) alongside a larger subgroup with the less severe form, which is premenstrual syndrome (PMS; up to 25%) [10,11]. Estrus cycle-dependent aggression in female rats can thus be studied as a model for PMS/PMDD in humans, using, for example, a resident–intruder (R/I) paradigm [1,2]. This PMDD model has the advantage of being a natural model, which means that the endogenous steroid production naturally varies throughout the estrus cycle of the rat; however, the number of individual rats showing aggression/irritability is higher than it is among humans [1,2]. Furthermore, hormone-related changes in aggression or irritability can be studied using this model, and these are major symptoms in PMDD.

Endogenous steroid-PAMs have a balancing role, and their effect on the GABA-A receptor ranges from agonistic to antagonistic [5,12]. Isoallopregnanolone (ISO; 3β-OH-5α-pregnan-20-one) is a 3β-hydroxy isomer of ALLO. It was previously shown that ISO is a GABA-A-modulating steroid antagonist (GAMSA), as it can antagonize the modulating effect of ALLO on the GABA-A receptor. This was determined in both in vitro studies of receptor effects [13,14,15] and in vivo studies of the effect on ALLO-induced anesthesia [16]. Unaccompanied by ALLO, ISO has no effect on GABAs’ effect on the GABA-A receptor, and it does not antagonize the effect of GABA, benzodiazepines, or barbiturates [13,17] One aim of this study was to evaluate the effect of ISO on diestrus aggression in female rats with estrus cycle-dependent aggression, which has, to our knowledge, not been previously studied. This is of interest as lately it has been shown that the blood ratio of ALLO/ISO concentrations relates to the activity in regions of emotional processing in women with PMDD, namely, the parahippocampal gyrus and amygdala different from controls [18].

The GABA-A receptor can appear in many different subunit forms. The most common sub-form is a receptor containing α1β2γ2L GABA-A receptor subunits [12]. Another subtype that has been extensively discussed in relation to animal models of PMDD is the α4βxδ GABA-A receptor subtype [19,20,21]. This subtype has been shown to relate to negative reactions in PMDD animal models, especially the expression of α4βxδ in the hippocampus [20,21,22,23,24]. The effect of ISO on extra-synaptic GABA-A receptors, and particularly the α4β3δ GABA-A receptor subtype, has not been previously shown. We have, therefore, investigated the effect of ISO on the THDOC modulation of human α4β3δ and show, as a comparison, the effect on α1β2γ2L GABA-A receptors expressed in HEK293-cells.

## 2. Materials and Methods

### 2.1. In Vitro Experiments

#### 2.1.1. Transfection of HEK-293 Cells for Electrophysiological Measurements

Wild-type HEK-293 cells passage 4, originally from the Pasteur Institute (Paris, France), were used for transfections. HEK-293 cells were permanently transfected with cDNA to express human α1β2γ2L and α4β3δ GABA-A receptor subtypes, respectively. The development procedure was described earlier for human α1β2γ2L and α4β3δ GABA-A receptor subtypes [25,26]. The transfected cells used for electrophysiological patch clamp experiments underwent a minimum of one passage after defrosting and were used 3–5 days after seeding. Cells were detached using trypsin and incubated for 15 min at 37 °C in extracellular (EC) solution (in mM: 137 NaCl, 5.0 KCl, 1.0 CaCl_2_, 1.2 MgCl_2_, 10 HEPES and 10 glucose; pH 7.4), The cells were seeded for a maximum of 10 times. The method for production of plasmids used for the sub-cloning of different subunits has been published earlier [27]. The cell lines produced were analyzed for the three GABA-A receptor subunits using immunocytochemistry (data not shown), followed by the selection of a suitable cell line showing good reactivity to GABA and THDOC.

#### 2.1.2. Electrophysiological Recordings

Electrophysiological recordings from HEK-293 cells were obtained via the whole-cell voltage clamp technique at room temperature (21–23 °C). After compensating for the liquid junction potential, a steady holding potential of −17 mV was used in all the experiments. Patch pipettes were made of borosilicate glass (Bergman labora AB, Danderyd, Sweden) and polished to a resistance of 2–5 MΩ when filled with intracellular solution and immersed in bath (extracellular) solution (for solutions see below). Recordings were made using an Axopatch amplifier, Digidata (Axon instruments, Foster City, CA, USA), controlled via a computer. Data were acquired using pCLAMP software, sampled at 10 kHz, filtered at 2–10 kHz, and analyzed using Clampfit (Axon Instruments, Foster City, CA, USA). A series resistance between the pipette and cell membrane of less than 20 MΩ was accepted. No resistance compensation was used. The stability of series resistance was monitored repeatedly during the experiments. The measured liquid junction potential between the extracellular and intracellular solutions was subtracted from all data presented, as previously described [17]. HEK-293 cells placed in a bath filled with extracellular (control) solutions were visually guided and patched using an inverted Zeiss Axiovert 25 microscope equipped with ×20 objective. In all experiments, the Dynaflow™ system (see below) was exploited for the quick and precise application of control and test solutions to the patched cells.

#### 2.1.3. GABA-A Receptor Pharmacology

**Figure 1:**
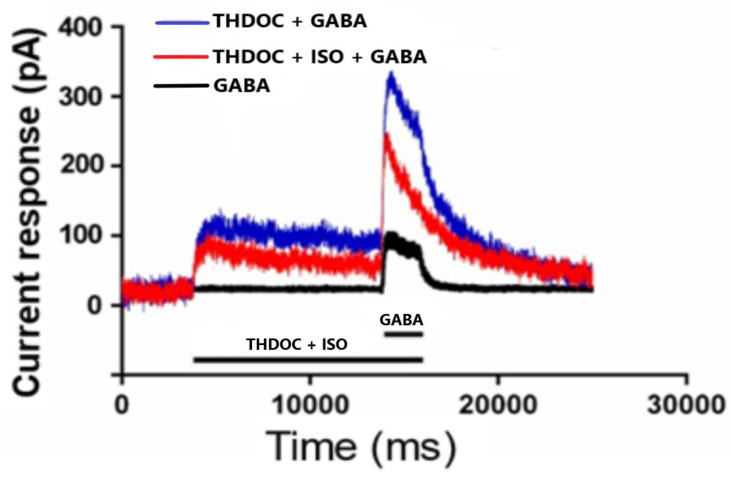
Responses (picko ampere, pA) in the human extra-synaptic GABA-A receptor α4β3δ expressed in HEK-cells. THDOC = 100 nM; ISO = 1 μM; GABA = 1 μM. Black line = GABA alone; blue line THDOC ± GABA; red line THDOC + ISO ± GABA.

Whole-cell recordings were taken under voltage clamp conditions in the chip bath (Dynaflow Resolve, Fluicell AB, Gothenburg, Sweden). Patch electrodes (1.5–4 MΩ) were filled with intracellular solution (in mM: 140 Cs-gluconate, 3.0 NaCl, 1.2 MgCl_2_, 10 HEPES, 1.0 EGTA and 2 MgATP; pH 7.2). THDOC (Sigma Chemical Co., St. Louis, MO, USA) and ISO (UC1010, 3α-ethynyl, 3β-hydroxyl, 5α-pregnane-20-one and sepranolone, Asarina Pharma AB, Stockholm, Sweden) were dissolved in dimethyl sulfoxide (DMSO) for the preparation of solutions containing a final DMSO concentration of 0.1% diluted in all solutions. Different protocols were used for different electrophysiology measurements, as the GABA-A receptor subunit combinations studied are present in the different parts of neurons. As α1β2γ2L- GABA-A receptors are intrasynaptic in vivo, experimental conditions resembling that situation, with the quick application (40 ms) of a high GABA concentration (30 μM). In contrast, α4,β,δ-GABA-A receptors are present extrasynaptically in vivo, and thus, the experimental conditions used were long exposures (6 s) to a low GABA concentration (0.3 μM) either in the absence or presence of THDOC and/or ISO [8 Carver and Reddy 2013]. For both cell types, the EC75s of THDOC were used. Cells were pre-exposed to THDOC alone or to THDOC + ISO 20s before GABA application. In each cell, the effects were normalized to the control response, and the area under the curve was analyzed. In all electrophysiological experiments, tetrahydro-deoxycorticosterone (THDOC) was used as a positive GABA-A receptor steroid modulator [5]. To evaluate the direct effect of neurosteroids on the GABA-A receptor-mediated response, HEK-293 cells were challenged with THDOC alone or in combination with ISO for 20s prior to and during GABA application (Figure 1). The application of all tested solutions was followed by at least 2 min of washing with a control solution.

#### 2.1.4. Dynaflow™ System

The Dynaflow™ system (Dynaflow Pro II Platform Zeiss Axiovert 25; Fluicell AB, Sweden) with Resolve chips was used for all patch clamp experiments. A Resolve chip consists of a glass chip enclosed by a PEEK plastic top part and a supporting plastic bottom part. Both the wells and the channels are glass coated. The channel width is 150 μm, and the height is 50 μm. The well volume is 150 μL. The run time at a flow rate of 26 μL/min is 90 min. The pump settings were as follows: a BD Plastikpak^TM^ 2 mL syringe with an inner diameter of 8.1 mm was used. The Resolve chip allows the synchronized control of switching between 16 experimental solutions. The laminar flow at each solution outlet of the microfluidic chip prevents mixing, and a computer-controlled stage motor is used to move the chip relative to the patch pipette, allowing rapid solution exchange around the membrane patch.

### 2.2. Data Analysis

Data analysis was performed, and graphical presentations were created using the Clampfit^TM^ data analysis module (Molecular Devices, LLC, Sunnyvale, CA, USA), the SPSS statistical package (IBM Corp., Armonk, NY, USA), Microsoft Excel (Microsoft Corp., Redmond, WA, USA) and the Origin package (OriginLab Corp., Northampton, MA, USA). Dose–response curves, EC_50_, IC_50_ and IC_max_ values were analyzed using GraphPad Prism software (GraphPad 6 Software Inc., La Jolla, CA, USA). Logistic equations were analyzed offline using Clampfit. The peak current and area under the curve within 20 ms (α1β2γ2L) or 10 s (α4β3δ, Figure 1) time windows were detected for each response. The mean baseline current during the 5 s interval preceding the inflection point when the current was evoked was considered as a reference point for the measured parameters. Currents with an amplitude of less than 10 pA were discarded from analyses. Data are presented as mean ± SEM, unless stated otherwise. The Kruskal–Wallis test and Wilcoxon matched-pairs signed-ranks test were used for statistical evaluation, with effects being considered as significant when *p* ≤ 0.05 (*), *p* ≤ 0.01 (**) and *p* ≤ 0.001 (***).

### 2.3. In Vivo Experimental Set-Up

**Figure 2:**
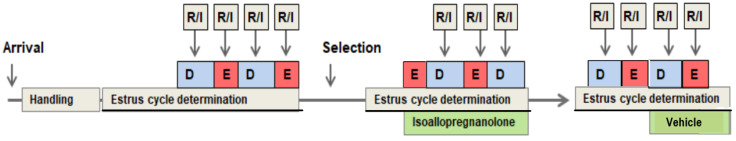
The study paradigm included estrus cycle phase determination and the investigation of estrus cycle-dependent aggressive behavior using resident/intruder (R/I) tests. R/I test were performed at both estrus (E) and diestrus (D) to determine estrus cycle-dependent aggression, i.e., the presence of aggressive behavior at diestrus and absence at estrus and during ongoing treatments with Isoallopregnanolone or a vehicle, to investigate the effect of ISO and vehicle on diestrus aggressive behavior.

The estrus cycle-dependent aggression of rats was investigated using a resident/intruder (R/I) test with behavioral analysis at diestrus and the estrus phases of the estrus cycle (Figure 2). The rats that showed estrus cycle-dependent aggression at the baseline, which indicates both the presence of aggressive behavior at diestrus and the absence of the same behavior at estrus, were selected for the treatment with ISO. The levels of aggressive behavior at diestrus and estrus during treatment were compared to those of their behavior prior to treatment. The intruder rats were ovariectomized animals of the same age and size as the residents.

#### 2.3.1. Animals

In total, 42 female Wistar rats (Taconics, Lille Skensved, Denmark) were used. Upon their arrival, the animals were approximately 9 weeks old, and they weighed 185–209 g. For identification, the rats were marked on their tails with permanent ink (PorciMark^TM^). The animals were housed in triads at the animal facility where all the experiments took place (Umeå University, Umeå, Sweden). A reversed light/dark cycle was used (12 h/12 h light/dark; lights off at 04:00). Food (standard chow) and water were available ad libitum. The rats were handled daily for two weeks prior to the start of testing. Nine rats were randomly selected as intruder rats and were ovariectomized. The remaining intact rats were tested for estrus cycle-dependent aggression, and 14 showed an aggressive behavior pattern. From the group of rejected rats, 12 were randomly selected for a satellite pharmacokinetic study (see below). The study protocol was approved by the Regional Ethics Committee of Umeå, Sweden. All animal handling and experiments were performed according to Swedish legislation.

#### 2.3.2. Ovariectomy of the Intruder Rats

The ovariectomy of the intruder rats was conducted via the ligation of uterine tubes and vessels and the removal of ovaries under anesthesia with 2.3% Isoflurane (Baxter Medical AB, Kista, Sweden) in oxygen (O_2_ flow rate: 0.32 L/min). The surgical suture techniques included the use of surgical glue (Vetbond Tissue Adhesive No 1469 3M) in addition to sutures. Pain relief (Rimadyl Vet, 0.25 mg/100 g) was given daily for one-week post-surgery.

#### 2.3.3. Determination of Estrus Cycle Phase

Estrus cycle phases (proestrus, estrus, metestrus and diestrus) of the rats were determined via the microscope examination of vaginal smears. The smears were collected daily between 08:00 and 10:00 using a glass pipette with 50–70 μL normal saline (NaCl 0.9%) and stained with 0.2% toluidine blue, and the cycle phase was identified according to Hubscher et al., [28] and Marcondes et al., [29]. However, on the day during which an R/I test was conducted, the vaginal smear of a tested animal was collected immediately after the encounter. In addition, during the treatments, smears were collected directly in the morning before injections to confirm the expected estrus cycle phase.

#### 2.3.4. Investigation of Estrus Cycle-Dependent Aggressive Behavior

Estrus cycle-dependent aggression was investigated using an R/I test that was similar to those described by Schneider and Popik [1] and Ho [2], but without the induction of pseudopregnancy. The R/I test was performed under dim white light during the animals’ dark active period (between 10:00 and 14:00) in a room that was similar and adjacent to their housing room. The resident rats remained in their home cages, while their cage mates were removed. After approximately 15 minutes’ habituation, an intruder was introduced for a period of eight minutes, and the encounter was recorded with a digital video camera (Sony HDR-CX360E) for later analysis. The intruder rats were marked using colorful felt tip pens for easy identification during video film analysis. The R/I test was performed at the diestrus and estrus of two baseline estrus cycles, which is four tests in total, to select individuals displaying estrus cycle-dependent aggressive behavior (Figure 2). In previous work using the similar animal model, no signs of habituation during the procedure were noted [1,2]. During the ISO treatment, aggressive behavior was studied via R/I tests during diestrus and estrus phases. After a pause in treatment, R/I tests were conducted at diestrus and estrus phases during untreated cycles and during the vehicle treatment.

#### 2.3.5. Video Film Analysis of Behavior

The behavior of the resident rat was quantified using Soldis SCORE software, version 3.4 (www.soldis.se/company_news.htm (accessed on 26 May 2023), Uppsala, Sweden), over five minutes from start of the intrusion (eight minutes in total). The operator was blinded to the cycle phase and the treatment of each rat, and the behaviors of the intruding rat were not analyzed. The analyzed parameters were attack frequency (aggressive outbursts towards the intruder, not including bites), the duration of non-aggressive social behavior (non-aggressive investigation of the intruder: sniffing, grooming, etc.) and the frequency of bites and mounting. Estrus cycle-dependent aggression was confirmed when a rat showed both presence of aggressive behavior at diestrus and the absence of the same at estrus during the tested estrus cycles.

#### 2.3.6. Treatment

The rats that displayed estrus cycle-dependent aggression were selected for the treatment phase of the study. The treatment (*n* = 14) was started at estrus and was composed of daily s.c. injections of 1.5 mg/kg ISO (5 mg/mL suspension in MCT oil; Asarina Pharma AB, Stockholm, Sweden) until the second diestrus phase had passed. The vehicle treatment (*n* = 7), which was given after the treatment was paused, used the same volume of MCT oil. After a non-treatment period, the vehicle treatment started during the estrus phase and ended when the second estrus had passed. The rats were tested using the R/I model (as above) during treatments given at diestrus and at estrus (Figure 2). The daily injections were given in the morning, and R/I testing took place 2 h afterwards.

#### 2.3.7. Pharmacokinetic Study

Twelve rats selected for pharmacokinetic analysis received the treatment above. The syringe with ISO suspension was weighed before and directly after the injection for verification of the given dose. Blood samples were collected on the third day of treatment at a specific time point 0 (i.e., just before the daily injection) and at 2, 4 and 8 h after the third injection. Due to there being a limitation in the volume of blood that can be withdrawn from each rat, at least samples from five rats were taken at each time point, and samples were taken on at least two occasions per rat. The samples were collected for plasma analysis according to the manufacturer’s instruction (BD Microtainer^®^ PSTTM LH tubes, BD, Franklin Lakes, NJ, USA). Plasma was immediately frozen and stored at −20 °C until it was shipped for analysis. ISO and ALLO were determined via LS-MS/MS, which is liquid chromatography-tandem mass spectrometry, employing the 2-hydrazino-1- methylpyridinium chloride derivatization of steroids and positive electrospray ionization mass spectrometry (OncoTargeting AB, Uppsala, Sweden). STable 2H- and ^13^C-labeled ISO were used as the internal standard, and the limit of quantitation was 0.3 ng/mL.

#### 2.3.8. Statistics

Statistical analyses were performed using SPSS 26 statistics software. One-way analysis of variance (ANOVA) with repeated measures was used to investigate the mean level of behaviors across the estrus cycle phases. The test for within-subject’s effect was performed first, the sphericity assumed test, and the second one that was performed was (when Mauchly’s test of sphericity was significant) the Greenhouse–Geisser test. *p* values below 0.05 were considered to be statistically significant, and the least significant difference test was used for ad hoc analysis when the overall ANOVA showed significance.

Among the individuals displaying estrus cycle-dependent aggression, the estrus and diestrus phases of the two cycles prior to treatment were analyzed to establish baseline behaviors. Then, the estrus and diestrus phases tested during treatments were compared to mean values of the pretreatment estrus and diestrus phases, respectively.

## 3. Results

### 3.1. In Vitro Experiments

In the α1β2γ2L GABA-A receptor subtype, ISO antagonizes THDOC + GABA enhancement in a concentration-dependent way.

**Figure 3:**
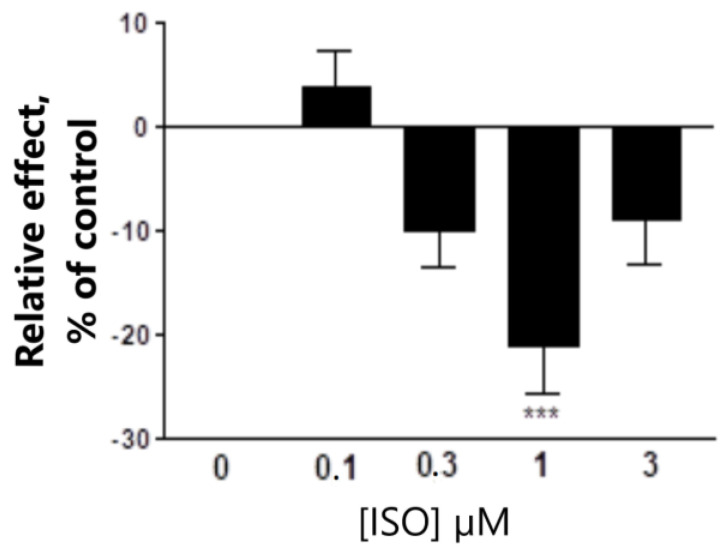
The effect of ISO (UC1010) concentration, 0.1 to 3 μM, in presence of 200 nM THDOC and 30 μM GABA in GABA-A receptor α1β2γ2L. Control, 200 nM THDOC + 30 μM GABA, was set to 0. *** *p* = 0.001.

In our setting with recombinant human α1β2γ2L, GABA-A receptor in HEK-cells THDOC +30 μM GABA dose dependently increase the current response up to maximum of 115% (Table 1). THDOC alone had no direct effect on the current response in the dose range used in this study. One μM ISO antagonizes the THDOC effect, but it does not affect the GABA response. In presence of 30 μM GABA, 1 μM ISO antagonizes the 200 nM THDOC-enhanced effect by −22.3 ± 5.3% (*p* < 0.001, *n* = 10). The modulatory effect of 1 μM ISO in the presence of 30 μM GABA alone was not significant (+10.3 ± 7.8%; NS, *n* = 9). This shows that 1 μM ISO had no significant effect on the GABA modulation of the GABA-A receptor. ISO in the concentration interval 0.1–3 μM was tested in the presence of 200 nM THDOC and 30 μM GABA. At the concentration of 0.3–3 μM, ISO significantly antagonizes the THDOC-enhanced effect, without completely blocking the effect (Table 1). ISO acts in a biphasic way with a maximal antagonizing effect, −21 ± 4.6% (*p* < 0.001; *n* = 10), at 1 μM ISO (Figure 3). From the best fit of the Hill equation, I_max_ and IC_50_ were calculated. I_max_ of the best fit curve was −21% of control (200 nM THDOC + 30 μM GABA = 0), and EC_50_ = 300 nM of ISO.

#### 3.1.1. The Effect of THDOC + GABA and ISO on α4β3δ GABA-A Receptor Subtypes

**Figure 4:**
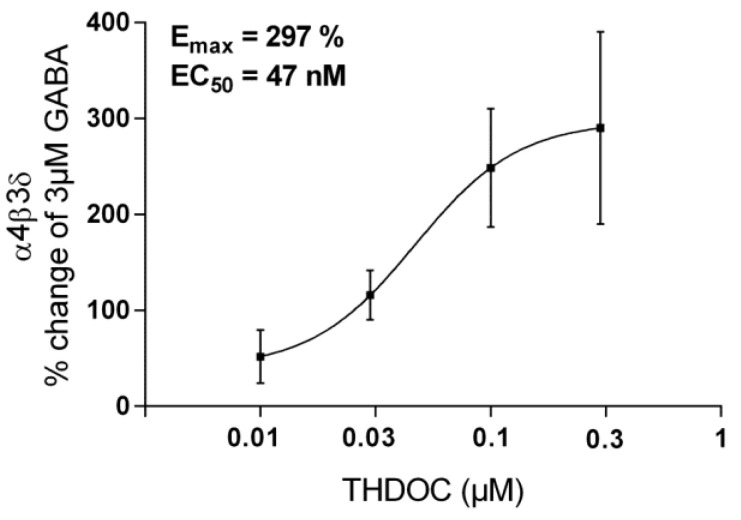
TA total of 0.01–0.3 μM THDOC enhanced the 3 μM GABA-mediated current in a concentra-tion-dependent way for the α4β3δ GABA-A receptor.

THDOC enhanced the current response together with GABA in our setting with recombinant human α4β3δ GABA-A receptor in HEK-cells. THDOC + 3 μM GABA dose dependently increase the current response up to maximum at 0.3 μM THDOC (E_max_ = 297%, EC_50_ = 47 nM; Figure 4). The EC_75_ of 0.1 μM THDOC was used as enhancer of 3 μM GABA when the effect of ISO was studied. A total of 1 μM ISO reduced the 0.1 μM THDOC enhanced effect by −26 ± 2.4% (N = 13; P = 0.001) (Table 2).

#### 3.1.2. The Effect of THDOC Alone, with or without ISO at α4β3δ GABA-A Receptor Subtype

THDOC alone (0.03–1 μM) more significantly induced the current response in the absence of GABA compared to that of the baseline (current response at only EC solution, Table 3). Studied were the effects of 1 μM ISO on the 0.1 μM THDOC-induced current response among the cells. The current response induced of 0.1 μM THDOC was 10 pA or more. A total of 1 μM ISO reduced the 0.1 μM THDOC-induced current response by −43 ± 3.5% (N = 11; P = 0.003) (Table 2). ISO (1 μM) alone had no effect on the α4β3δ GABA-A receptor (0.4 ± 0.3 pA; N = 11, NS) compared to that of the current response when the EC solution was used (−0.1 ± 0.3 pA; N = 10; NS).

### 3.2. In Vivo Experiments

#### 3.2.1. Baseline Behavior

**Figure 5:**
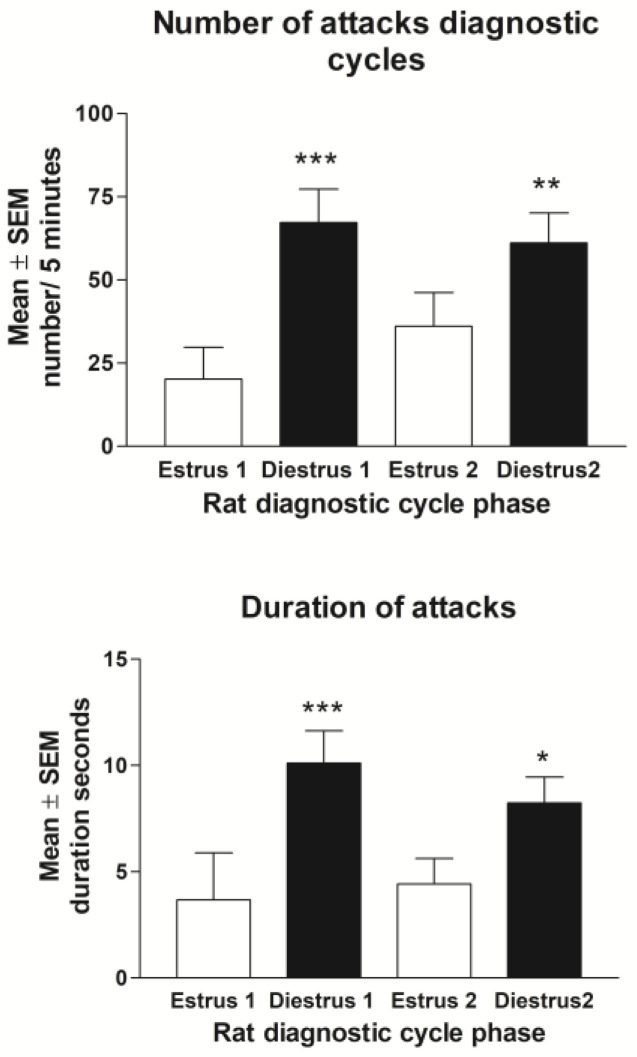
Baseline estrus cycle-dependent aggression during two untreated cycles, respectively. The aggressive behavior level is elevated at diestrus compared to that at estrus. Top data show mean ± SEM of number of attacks by the resident rats (N = 14) towards the intruders during the first five minutes of the intrusion (*** *p* < 0.001 vs. estrus cycle 1. ** *p* < 0.01 vs. estrus cycle 1). Bottom data show duration of attacks of resident rat against intruder (*** *p* < 0.001 vs. estrus cycle 1. * *p* < 0.05 vs. estrus cycle 2).

Fourteen (42%) of the thirty-three tested rats showed estrus cycle-dependent aggression in at least one of the two tested cycles. This is higher than it is for women who have PMDD [11]. In this selected group (*n* = 14), the attack frequency was significantly higher at diestrus than it was at estrus (F(3,36) = 8.131; *p* < 0.001; Figure 5). The ad hoc test revealed that at the group level, the difference between the estrus phases was significant in the first cycle (*** *p* < 0.001), but it was not in the second one (*p* = 0.09). However, the level of aggressive behavior during the second diestrus was significantly elevated compared to that of the first estrus (** *p* < 0.01), and no differences were found between the two estrus phases or the two diestrus phases, respectively. The duration of aggression showed a similar pattern, with significantly longer aggression periods in diestrus compared to those in the estrus phases in the same cycle (*p* < 0.001 and *p* < 0.05).

**Figure 6:**
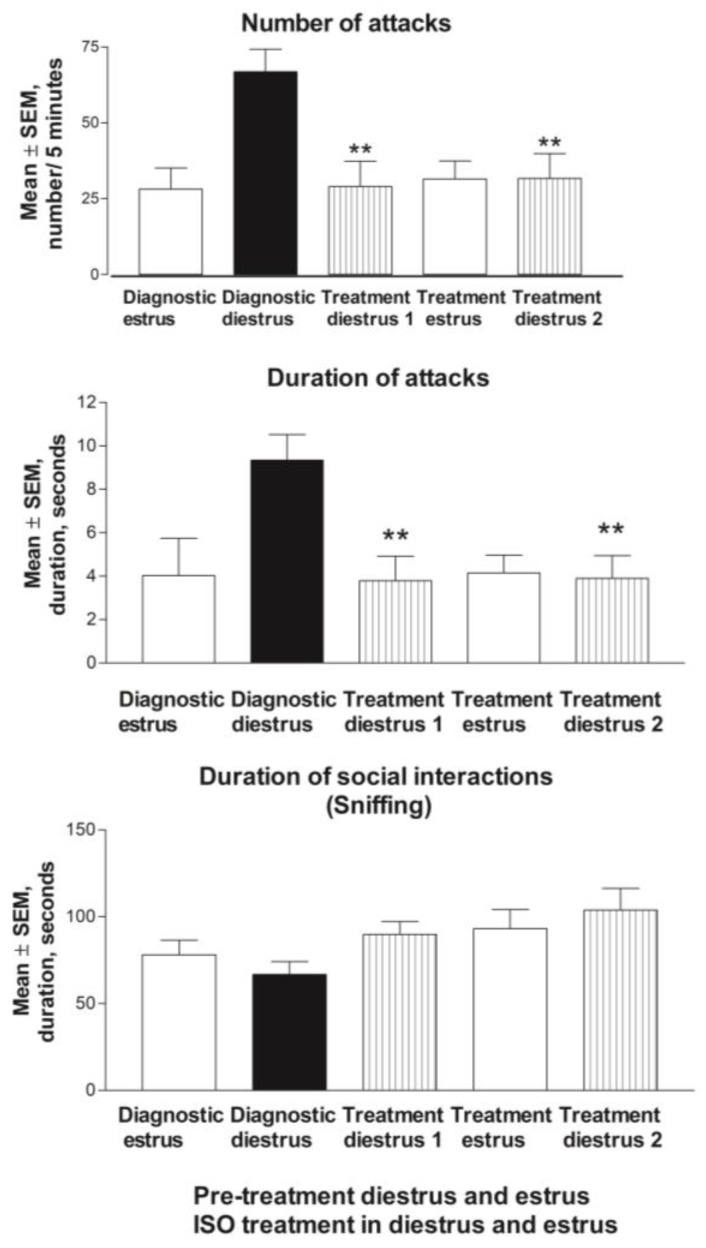
Estrus cycle-dependent aggression during ISO treatment compared to that of mean baseline. Top picture data show mean ± SEM of number of attacks during the combined two pretreatment cycles and the two ISO treated cycles by the resident rats towards the intruders during the first five minutes of the intrusion. The aggressive behavior level at diestrus is diminished during ISO treatment compared to that of the baseline. ** *p* < 0.01 vs. diestrus baseline. No significant difference between diestrus and estrus phases. Middle figure shows duration of attacks, and bottom figure shows duration of social interactions (sniffing).

The analysis of phase-specific mean values of the two cycles combined revealed that the attack frequency was significantly higher at diestrus compared to that at estrus (65.2 ± 7.2 attacks ± SEM vs. 26.0 ± 6.7; *p* < 0.001). (Figure 6). Biting and mounting behaviors were observed only sporadic, and therefore, were not included in statistical analyses.

Nine (27%) of the thirty-three rats showed estrus cycle dependent aggression in both tested cycles. In this group, aggressive attacks were more frequent at diestrus than they were at estrus (F(3,24) = 14.086; *p* < 0.001), but they were evident in both cycles, respectively (1st cycle *p* = 0.003, and 2nd cycle *p* = 0.003). The level of non-aggressive interaction (sniffing behavior) did not differ between the tested estrus cycle phases (Figure 6).

#### 3.2.2. Estrus Cycle and Behavior during ISO Treatment

Fourteen rats who showed estrus cycle-dependent aggression were selected for the treatment phase of the study. The estrus cycle duration or vaginal smear’s appearance did not change during the ISO treatment (data not shown). The displaying of aggressive behavior at diestrus during treatment with ISO was compared to the mean value of everyone’s aggressive behavior during diestrus at the baseline. When the rats were treated with ISO, the attack frequency was significantly decreased at diestrus during the treatment in comparison to that of the mean diestrus result when the treatment was not given (F(2,26) = 7.127; *p* = 0.003; Figure 6). A decrease in attack frequency and duration of attacks at diestrus was observed in both tested cycles, which was confirmed via ad hoc analysis (1st cycle *p* < 0.01, and 2nd cycle *p* < 0.01). No differences in attack frequency were found between estrus during treatment and estrus at the baseline. More importantly, no differences in attack frequency were found between the two diestrus phases during the treatment and either estrus during the treatment or estrus at the baseline. The level of non-aggressive interaction (sniffing behavior) did not differ between the tested estrus cycle phases (Figure 6).

To calculate the relative effect of the ISO treatment on estrus cycle-dependent aggressive behavior at diestrus, the relative effect on aggressive behavior at diestrus was calculated. When the baseline diestrus aggression level was set to 100%, and the baseline estrus aggression level was set to 0%, the effect of the ISO treatment was −89%, and the effect size, which was calculated as Cohen’s d, = 1.67.

The group of nine rats that showed estrus cycle-dependent aggression in both tested pre-treated cycles were tested on four tests’ occasions: (a) individual baseline mean diestrus attack frequency (b) diestrus 1, (c) estrus and (d) diestrus 2. The aggressive attacks were less frequent at both diestrus phases when they were treated with ISO compared to that at diestrus at the baseline (F(4,32) = 7.820; *p* < 0.001, ad hoc: *p* = 0.044 and *p* = 0.005, respectively). The levels of aggressive attacks at diestrus during the treatment were not different compared to either estrus during the treatment or estrus at the baseline, and there was no difference between the estrus phases. The level of non-aggressive interactions (sniffing behavior) did not differ between the tested estrus cycle phases (Figure 6).

#### 3.2.3. Estrus Cycle and Behavior during Vehicle Treatment

**Figure 7:**
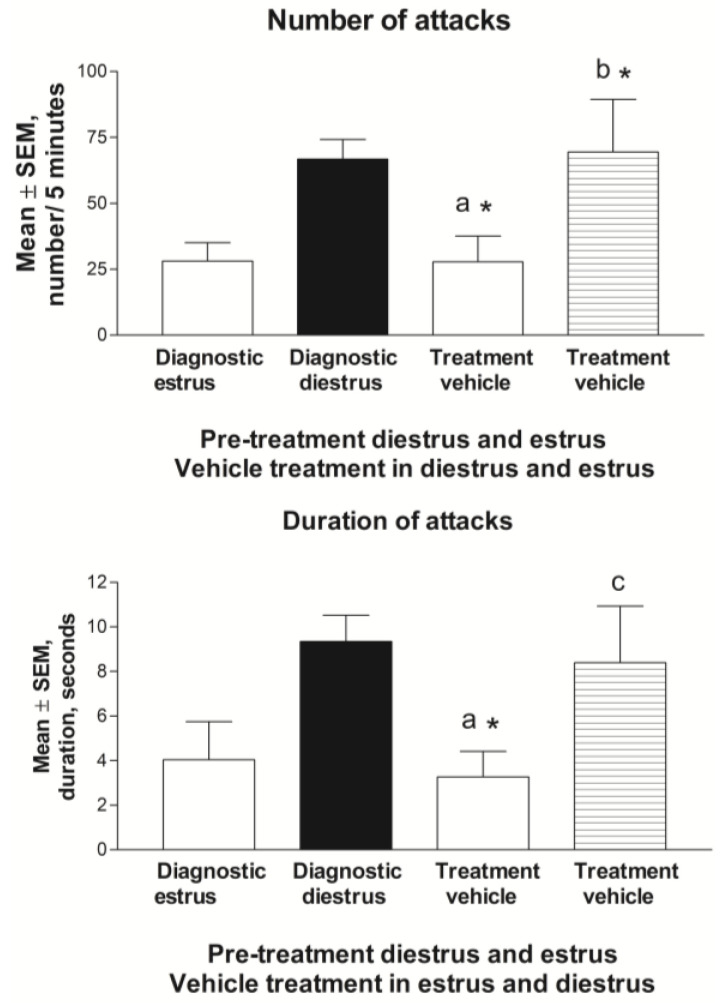
The number of attacks and duration of attacks during pretreatment and during one cycle of vehicle treatment are depicted. During the vehicle-treated estrus phase, the number of attacks were significantly lower (a, * *p* < 0.05) compared to that during the pretreatment diestrus phase and were at the same level (*p* = NS) as that during the pretreatment estrus phase. During the vehicle-treated diestrus phase, the number of attacks was significantly higher than that during the vehicle-treated estrus phase (b, * *p* < 0.05), and the diestrus duration of aggression showed a trend in the same direction (c, *p* = 0.061).

The treatments given to the rats were paused for two weeks before the crossover to the vehicle treatment was performed. Seven of the rats showed similar behaviors as they did in the pretreatment period and were considered to have recovered. The rats were given vehicle injections in the same way as they did during the active treatment, as described in the Methods Section. The aggressive behavior had the same pattern as that which was seen during the pretreatment, which is more aggressiveness in diestrus compared to that at estrus (*p* < 0.05), even though there was a greater variation compared to that during the pretreatment period (Figure 7).

#### 3.2.4. Plasma Concentrations of ISO and ALLO

**Figure 8:**
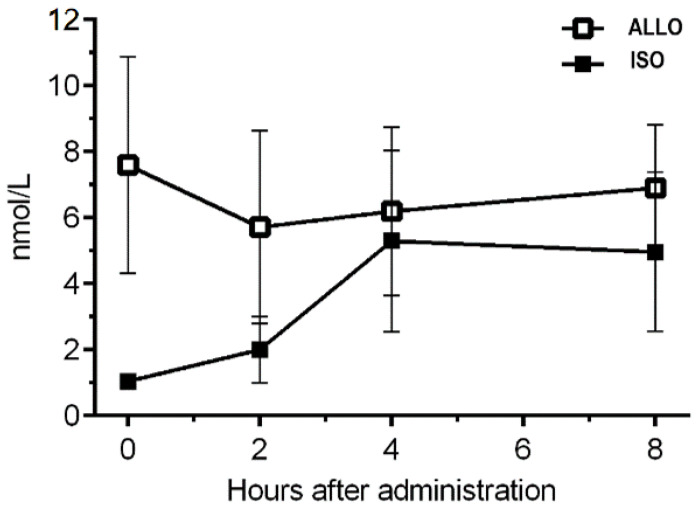
Plasma concentration (nM) of ALLO and ISO at the third day of ISO treatment (s.c. 1.5 mg/kg in a 5 mg/mL oil-based suspension).

The plasma ISO levels during treatment were lower than the endogenous ALLO levels were (Figure 8). The highest level of ISO was measured four hours after injection. ISO reached an approximate five-fold increase, and it nearly reached the level of ALLO, resulting in an ISO:ALLO ratio of 1:1. At the time, when the R/I test was conducted, two hours after the injection, the ISO level was approximately doubled compared to that before the injection, but it was only a third as high as the endogenous ALLO level, resulting in an ISO:ALLO ratio of 0.3:1.

## 4. Discussion

In this study, we replicated previously reported results by showing that a subgroup of female rats displayed a cyclical pattern of aggression, which was dependent on their estrus cycle phases [2]. More importantly, we found that estrus cycle-dependent aggression at diestrus was alleviated by a treatment with the endogenous GABA-A-modulating steroid antagonist (GAMSA), isoallopregnanolone (ISO). The therapeutic effect was seen in a group of rats that were confirmed to show estrus cycle-dependent aggressive behavior. During the exposure to ISO, the level of aggression at diestrus was equivalent to that at the baseline estrus period, when the aggression level was minimal. The level of aggressive behavior at estrus was, in general, low, and it was not changed via the application of the ISO treatment in comparison to that at the baseline. During the vehicle treatment, the cyclicity returned to a similar pattern with increased aggression during the diestrus phase, such as that seen in the estrus cycle during the pre-treatment phases. The results indicate that ISO has an aggression-dampening effect in female, normally cycling rats. Additionally, we found that the rats’ non-aggressive behavior was not affected by the ISO treatment, as the level of social interaction remained unchanged throughout the whole experiment.

In this study, we also show that ISO has an antagonistic action on the THDOC-enhanced effect on GABAs’ current response for both the α1β2γ2L and α4β3δ GABA-A receptors. In other rat models of PMDD, the α4βxδ receptor subtype has been determined as being responsible for irritability behavior and associated with signs of negative moods [20,21,22,23,24,30]. In these studies, ISO has been shown to specifically antagonize GABA-A receptor-modulating steroids at the α4β3δ GABA-A receptor, but it does not antagonize GABA itself, and it has been previously shown that ISO does not antagonize barbiturates or benzodiazepines [13,17]. In vivo studies with ISO have been previously shown to antagonize ALLO-induced anesthesia, although in much higher dosages [16]. ISO has also been shown to cause an allopregnanolone-induced reduction in saccadic eye velocity and sedation in humans [31]. ISO has, however, not earlier been tested in a model of premenstrual dysphoric disorder (PMDD), such as in the present study.

The resident–intruder (R/I) testing of estrus cycle-dependent aggression is considered to be an animal model for sex steroid-related negative mood symptoms or for human premenstrual disorders [2]. PMS, and especially the more severe form, PMDD, is believed to be caused by the paradoxical effect of moderately increased ALLO levels that occur during the luteal phase in the human menstrual cycle or at diestrus in the rat estrus cycle [3,8,32,33,34]. Higher levels of ALLO, such as those that occur during late pregnancy or during rats’ estrus peak, seem not to induce the paradoxical effect. Actually, these high ALLO levels are calming and anxiolytic [8]. In humans, PMDD symptoms are alleviated in anovulatory menstrual cycles. This was shown both in the case of spontaneous anovulation and when it was induced by using GnRH agonists [35,36,37]. Similarly, it has been shown that ovariectomized rats do not show estrus cycle aggressive behavior at diestrus, and aggression returned when the rats were given progesterone and estrogen supplements to compensate for the ovariectomy [2]. Among postmenopausal women and among women with PMDD given a GnRH-agonist treatment, and given hormone replacement, symptoms are similar to those of PMS/PMDD during the progesterone phase of cyclical hormone replacement therapy [7,38]. Another option to reduce PMS/PMDD symptoms is a treatment with selective serotonin re-uptake inhibitors (SSRIs). Treatment with the SSRI fluoxetine has been studied in rats with estrus cycle-dependent aggression, and the number of rats that showed aggressive behavior at diestrus was significantly reduced [2]. In humans, an SSRI treatment has been shown to reduce symptom severity in PMDD. However, it comes with risks for side effects, such as nausea and sexual dysfunction, especially the orgasmic type [39]. The estrus cycle-dependent marble-burying regime is another animal model for PMS/PMDD, and rats shows increased marble burying behavior at diestrus in comparison to that at estrus. In this model, the treatment, including single injections of SSRI four hours before testing, significantly decreased the number of marbles buried at diestrus [40]. With a 10 mg/kg fluoxetine treatment, the level of marble burying decreased by 80%. Here, we showed that 89% of the estrus cycle-dependent aggressive behavior was alleviated with the 1.5 mg/kg ISO treatment. ISO is an endogenous neurosteroid and may have a natural role as an ALLO antagonist to balance GABA modulation in the central nervous system. Therefore, it is not surprising that ISO antagonized estrus cycle-dependent aggressive behavior in female rats. In this study, mainly aggression/irritability was investigated. These symptoms are key symptoms in PMDD; however, there are other important symptoms, such as depression and anxiety, that were not investigated using this rat model.

Neurosteroids and other positive GABA-A receptor-modulating compounds have a biphasic effect pattern. This means that a high level of exposure to these compounds has a calming and sedative effect, while moderately dosed exposure can lead to irritability and aggressive behavior, as shown in, for example, rodents [9,41]. In this study, the presence of ALLO was endogenous, and the levels of ALLO were at normal baseline levels. During the treatment with ISO, and at the time of testing, the ALLO levels seemed unaffected, and the ISO levels were approximately doubled compared to the endogenous baseline levels. This increase is modest and reveals that a low level of ISO is sufficient to antagonize estrus cycle-dependent aggressive behavior.

In this study, 42% of the tested population showed estrus cycle-dependent aggression in at least one of two estrus cycles, and 27% showed it in both cycles. This is comparable to previously reported data using the R/I model, where 30% of the rats displayed aggressive behavior at diestrus, which was compared to almost no anxiety behavior being seen at estrus [1]. In another study, a significantly higher incidence rate of 64% was reported [2]. However, Ho et al. subjected all rats to pseudopregnancy, which may have heightened the level of aggressive behavior. In this study, we found no differences in estrus phase behaviors between the diagnostic and the treatment cycles. This supports the hypothesis that the effect of ISO acts on hormonally induced effects during the diestrus phase and not on some basic behaviors. The fact that not all animals show the estrus cycle-dependent aggressive pattern is no surprise because, among humans, only 3–8% of women of fertile ages have severe premenstrual negative mood symptoms (PMDD) [10,11]. In addition, some individuals describe negative mood symptoms that consistently occur in every menstrual cycle, while others experience this only in some cycles. However, the DSM-V (and previous) diagnostic criteria for PMDD are strict and do not include only irritability or aggression; thus, the prevalence rates are lower. Still, our findings suggest that rats also show individual variation in occurrence consistency, which indicates that it is similar to that of humans.

In another study using the R/I model, it was reported that 40% of unmanipulated, young adult female rats showed aggressive behavior independent of the estrus cycle phase [42]. Interestingly, they also found that adolescent individuals with high-level anxiety displayed higher levels of aggression that was not alleviated by oxytocin, while individuals with low-level anxiety reacted positively to oxytocin. This would imply that, even though an estrus cycle variability was not confirmed, individual differences are determinants for the mechanisms behind aggressive behavior. These individuality traits may lie in the specific expression of GABA-A receptor subunits in each individual, leading to different responses to steroid-PAM exposure [12].

In conclusion, in our study, we show that ISO can antagonize the steroid-PAM THDOC in human α1β2γ2L and α4β3δ GABA-A receptors. We also conclude that the ISO treatment can antagonize the heighten the level of aggression during diestrus, possibly via inhibiting the effect of ALLO on the GABA-A receptor.

## Figures and Tables

**Table 1:** The current responses mediated via chloride ion flux through the GABA-A receptors expressing α1β2γ2L were studied via the use of the patch clamp technique combined with the Dynaflow™ application system and the Resolve chip. This provided rapid (~40 mS) applications of and the removal of substances.

Tested Dosages Combinations:	Effect Mean ± SEM% (N; P)
0.3–1 μM THDOC + 30 μM GABA (%) at α1β2γ2L	+115 E_max_% (N = 35, P = 0.001)
1 μM ISO + 30 μM GABA (%) at α1β2γ2L	+10.3 ± 7.8% (N = 9, NS)
1 μM ISO + 30 μM GABA + 0.2 μM THDOC at α1β2γ2L	−21 ± 4.6% (N = 10, P = 0.001)

**Table 2:** The current responses mediated via chloride ion flux through the expressed α4β3δ GABA-A receptor were studied via the use of the patch clamp technique combined with the Dynaflow™ application system.

The Effect of 1 μM ISO on	Mean ± SEM% (P; N)
1 μM ISO + 3 μM GABA response (%); at α4β3δ	−6.7 ± 3.3% (N = 11, NS)
1 μM ISO + 0.1 μM THDOC + 3 μM GABA (%); at α4β3δ	−26 ± 2.4% (N = 13, P = 0.001)
1 μM ISO + 0.1 μM THDOC, baseline (%); at α4β3δ	−43 ± 3.5% (N = 11, P = 0.003)

**Table 3:** A total of 0.3 μM to 1 μM THDOC significantly induced a current response in the absence of GABA at α4β3δ.

THDOC (μM) Baselineshift	Mean ± SEM pA (N; P)
0.03	+5 ± 3.3 pA (N = 9; P = 0.008)
0.1	+15 ± 9.5 pA (N = 9; P = 0.003)
0.3	+12 ± 7.9 pA (N = 9; P = 0.019)
1	+69 ± 35 pA (N = 3; P = 0.018)

## Data Availability

Data is unavailable due to patent issues.

## Declarations

ISO (UC1010) is owed by Asarina Pharma AB.
